# Short-Chain Fatty Acids and Palmitate Induce Distinct Metabolic and Phenotypic Signatures in Normal and Ischemic Skeletal Muscle Microvascular Endothelial Cells

**DOI:** 10.3390/cells15060493

**Published:** 2026-03-10

**Authors:** Andrew Guilfoyle-Speese, Kripa Patel, Aishwarya H. Ghanwat, David Stepp, Vijay Ganta

**Affiliations:** 1 Department of Physiology, Vascular Biology Center, Augusta University, Augusta, GA 30912, USA; aspeese@augusta.edu (A.G.-S.); dstepp@augusta.edu (D.S.); 2 Department of Medicine, Vascular Biology Center, Augusta University, Augusta, GA 30912, USA; kpatel33@augusta.edu (K.P.); aghanwat@augusta.edu (A.H.G.); 3 College of Science and Mathematics, Augusta University, Augusta, GA 30912, USA

**Keywords:** angiogenesis, barrier, metabolism, mitostress, glycostress, fatty acid oxidation

## Abstract

**Highlights:**

**What are the main findings?**

- Palmitic acid aggravates ischemic vascular dysfunction by decreasing endothelial cell survival, angiogenic capacity, barrier integrity, and overall metabolic health.
- Short-chain fatty acids enhance glycolysis–OxPhos coupling in ischemic endothelial cells to induce their angiogenic capacity with preserved barrier integrity.

**What are the implications of the main findings?**

- Palmitic acid could be a contributing factor to the disease severity observed in peripheral artery disease patients with diabetes.
- Short-chain fatty acids could be a potential therapeutic used to revascularize ischemic muscle in patients suffering from peripheral artery disease.

**Abstract:**

**Background**: Palmitate, a long-chain fatty acid, is well known to be a significant risk factor for cardiovascular diseases. In our current study, we wanted to determine whether palmitate treatment further aggravates ischemic endothelial cell (EC) injury and can serve as an in vitro model that emulates diabetic peripheral artery disease (diabetic-PAD). Short-chain fatty acid (SCFA) treatment was used as an additional comparator for palmitate-induced vascular dysfunction in normal or ischemic ECs in vitro. **Methods**: Hypoxia serum starvation (HSS) was used as an in vitro model for PAD. Cell survival or proliferation was determined by the CCK8 kit. EC angiogenic capacity was determined by in vitro tube formation assays on growth factor-reduced Matrigel. EC barrier integrity was determined by trans-endothelial electrical resistance measurements by EVOM3. EC metabolic phenotyping was performed by Seahorse glycolysis, mitochondrial respiration, and fatty acid oxidation metabolic assays. **Results**: Palmitate dramatically decreased the survival of normal and ischemic ECs, whereas SCFAs did not have a significant effect on ischemic EC survival. In vitro angiogenic assays showed that palmitate significantly decreased the angiogenic capacity of ischemic ECs, whereas SCFAs significantly induced their angiogenic capacity. While palmitate significantly decreased normal and ischemic EC barrier integrity, SCFAs improved normal and ischemic EC barrier integrity. Metabolic assays showed that palmitate significantly decreased normal EC mitochondrial respiration but not glycolysis. However, palmitate significantly decreased overall metabolic health, including mitochondrial respiration and glycolysis in ischemic ECs. On the contrary, SCFAs increased both mitochondrial respiration and glycolysis in normal ECs. In ischemic ECs, SCFAs induced mitochondrial respiration with a concomitant decrease in glycolysis. Fatty acid oxidation analysis showed that, unlike palmitate, which depends on carnitine palmitoyl transferases (CPTs) for β-oxidation in both normal and HSS ECs, SCFAs depend partly on CPTs to undergo β-oxidation in HSS ECs but not in normal ECs. **Conclusions**: While palmitate inhibits ischemic EC angiogenic capacity by decreasing overall metabolic health, SCFAs induce glycolysis–mitochondria OxPhos coupling to induce ischemic EC angiogenic capacity.

## 1. Introduction

Endothelial cells (ECs) are at the center of vascular remodeling processes that drive perfusion recovery in peripheral artery disease (PAD) [1]. The EC barrier not only limits fluid influx into the tissue but also prevents circulating fatty acids from depositing into the vascular wall [2]. Hence, controlling lipid/fatty acid levels is critical not only to mitigate atherosclerosis progression but also to reduce the cardiovascular disease (CVD) burden [3]. Based on the number of carbon atoms, fatty acids are sub-grouped into the following three types: short-chain fatty acids (SCFAs, ≤6 carbons), medium-chain fatty acids (8–12 carbons), and long-chain fatty acids (LCFAs, ≥14 carbons) [4]. LCFAs are further categorized into saturated and unsaturated fatty acids (monounsaturated or polyunsaturated) depending on the number of carbon double bonds. Palmitic acid, the most abundant saturated fatty acid, has been shown to induce EC inflammation and contribute to atherosclerosis [5]. In fact, a multivariate regression analysis from the Insulin Resistance Atherosclerosis Study [6] (IRAS) indicated that palmitate is a strong risk factor for type-2 diabetes (T2D) independent of insulin resistance.

SCFAs are the metabolic byproducts of dietary fibers (predominantly) produced by gut bacteria [7]. Acetate > propionate > butyrate (60:20:20 mM/kg) are the most abundant SCFAs produced in the colon with preferential oxidation toward butyrate > propionate > acetate by gut commensals for energy production [8]. In a clinical study by Muradi et al. [9], increased fecal excretion of acetate, butyrate, and propionate showed a weak positive correlation with random blood glucose levels, peak systolic velocity, volume flow, and plaque formation, indicating a higher cardiovascular risk in PAD patients with diabetes [9]. Endothelial dysfunction, characterized by reduced nitric oxide (NO) bioavailability, increased oxidative stress, leukocyte adhesion molecule up-regulation, and permeability changes, is a hallmark of cardiovascular diseases such as hypertension and atherosclerosis, as well as diabetes. The ability of SCFAs to modulate metabolic parameters implicated in EC dysfunction suggests a potential mechanistic link between gut microbial metabolites and vascular health. For example, dietary fiber intake (which boosts SCFA generation) may influence EC performance via metabolite-mediated signaling. Furthermore, targeting GPR41/43 activation or enhancing SCFA production might represent adjunctive strategies for maintaining vascular health.

While the β-oxidation of palmitate in mitochondria occurs via the carnitine–palmitate shuttle system [10], SCFAs can freely diffuse into the mitochondrial matrix and enter the β-oxidation due to their smaller sizes [11]. We have recently shown a detrimental role of palmitic acid in aggravating ischemic EC dysfunction [12]. However, the relative contribution of palmitate or SCFA substrate reliance on ischemic EC function remains largely unexplored. Hence, based on the emerging roles of SCFAs in regulating cardiovascular diseases [13], we compare the palmitate- vs. SCFA-mediated metabolic and phenotypic effects on ischemic ECs in vitro.

## 2. Materials and Methods

### 2.1. Cells and Cell Culture

The mouse skeletal muscle microvascular endothelial cell line [14] (mSkVECs) was purchased from Cell Biologics, Chicago, IL, USA (Cat No: C576220IM) and cultured in complete mouse endothelial cell medium supplemented with M1168 Kit (Cell Biologics, Cat No: M1168) [14].

### 2.2. Hypoxia Serum Starvation

Cells were incubated in an endothelial starvation medium (Cell Applications, Cat No: 209-250) and subjected to hypoxia (2% O_2_) for 24 h [12,14].

### 2.3. Cell Treatments

BSA, BSA-palmitate kit (Cat No: 29558, Cayman Chemicals, Ann Arbor, MI, USA), and water-soluble short-chain fatty acid mixture (Cat No: 28682, Cayman Chemicals) were dissolved in normal or HSS media. Cells were treated with BSA (*v*/*v*), BSA–palmitate (100, 250, or 500 µM), or SCFAs (100, 250, or 500 µM) under normal or HSS conditions according to the experimental requirements for 24 h, followed by downstream experiments. The pH was adjusted to 7.4 with 1 N NaOH.

### 2.4. Cell Proliferation/Survival

Normal ECs at 50% confluence or HSS ECs at confluence cultured in 96-well plates were treated under normal or HSS conditions for 24 h. Post-treatment, ECs were incubated with CCK8 reagent in a CCK8 cell counting kit (Cat No: K1018, ApexBio, SanDiego, CA, USA) for 1 h according to the manufacturer’s instructions. Post-incubation, the plates were colorimetrically read at 450 nM using a BioTek Synergy LX Multi-Mode Plate Reader (Agilent, Santa Clara, CA, USA. Each well in the 96-well plate was considered a biological replicate, and each assay was repeated twice.

### 2.5. In Vitro Angiogenesis (Capillary-like Loop Formation) Assay

Endothelial cells post-treatment were trypsinized, and the cell number was quantified using the Corning CytoSmart cell counter (Corning, Glendale, AZ, USA). An equal number of cells (~5000–7500) were plated on growth factor-reduced Matrigel (Cat No: AC-M082703, Acro Biosystems, Newark, DE, USA) to avoid any interference from VEGF-A present in the Matrigel. Our typical Matrigel assays employ ~25–30 K ECs/well [12,14]. This cell density allows clear tube-like structures on Matrigel similar to HUVECs. However, due to the magnitude of angiogenesis induced by SCFAs, we have decreased the number to 5–7.5 K/well, which otherwise resulted in tube-like structures stretching and falling apart on the Matrigel. While we were able to see the formation of branches, nodes, and junctions, this smaller number was not sufficient to make tube-like structures on the Matrigel. Since the primary question was to determine the role of SCFAs in promoting EC angiogenic capacity, we adhered to ~5–7.5 K cells/well in our assays. In summary, these mSKVECs form clear tube-like structures on Matrigel comparable to those of HUVECs. However, in our current study, we had to decrease the cell count to clearly see the angiogenic changes induced by SCFAs, which may have resulted in very minimal tube-like structures on the growth factor-reduced Matrigel. Furthermore, considering the manuscript by DeCicco-Skinner et al. [15], which suggests that the best measurement for angiogenesis is the nodes, followed by the junctions/branches and meshes, we excluded the quantification of meshes in our angiogenic assays in vitro. Capillary-like structures on the Matrigel in each well were photographed at 4–6 h at the center concave, and the numbers of nodes, junctions, and branches were quantified using NIH Image J (V2) Angiogenesis Analyzer. Each Matrigel well was considered a biological replicate, and the assay was repeated twice.

### 2.6. Trans-Endothelial Electrical Resistance (TEER, Ohms/cm^2^)

ECs were cultured in 8.0 µm transwell inserts. At confluence, ECs were treated according to the experimental requirements under normal or HSS conditions. The TEER of the barrier was measured using EVOM3 (World Precision Instruments, Sarasota, FL, USA). Data were plotted as the % change from the 0 h time point [16]. Each transwell insert was considered a biological replicate, and each assay was repeated twice.

### 2.7. Seahorse Metabolic Assays

mSkVECs were cultured in 6-well plates and at confluence were treated with palmitate (250 µM), SCFA (500 µM), and BSA (control) under normal or HSS conditions for 24 h. Post-treatment, cells were trypsinized and 30,000 live cells (assessed by trypan blue dye exclusion)/well were plated in Seahorse cell culture plates, centrifuged at 400× *g* for 5 min, followed by 1 h of equilibration and attachment in Seahorse base medium. Later, Seahorse XFe24 glycostress, mitostress and fatty acid oxidation (FAO) assays were performed in normal or HSS mSkVECs using 5 mM D-Glucose, 1 µM oligomycin and 50 mM 2-DG for glycolysis; 2.5 µM oligomycin, 5 µM FCCP and 0.5 µM rotenone/antimycin for mitostress; and 4 µM etoxomir, 2.5 µM oligomycin, 5 µM FCCP and 0.5 µM rotenone/antimycin (without palmitate in the assay medium) for FAO according to the standard Seahorse assays using Seahorse Xfe24 analyzer (Agilent, Santa Clara, CA, USA) [14,17]. Glycolysis, glycolytic capacity, glycolytic reserve in the glycostress assay, as well as basal respiration, maximal respiration, and ATP production in both mitostress and FAO, were normalized to the cell count after subtracting the baseline from wells that did not have any cells according to the standard Seahorse assay protocols. Each well was considered a biological replicate, and each assay was repeated at least twice.

### 2.8. Statistics

GraphPad Prism 9 was used to determine the statistical significance of the data and generate graphical representations. Data from biological replicates were analyzed for equal variance using the F test. Datasets that passed the F test were analyzed by the Unpaired *t* test for 2-group comparisons or with one-way ANOVA followed by Bonferroni’s or Dunnett’s post-test for the comparison of multiple groups. Welch’s correction was applied to the T-test analysis of the data that did not pass the equal variance test. Two-way repeated measures ANOVA with Bonferroni was used for the TEER measurements. Outliers were detected and removed by performing Grubb’s test. Statistical tests for each experiment are provided in the figure legend. *p* < 0.05 was considered significant in all the experiments [12,14,17]. Data are presented as the mean ± SEM.

## 3. Results

### 3.1. Unlike Palmitate, SCFAs Do Not Inhibit Ischemic EC Proliferation

**Figure 1:**
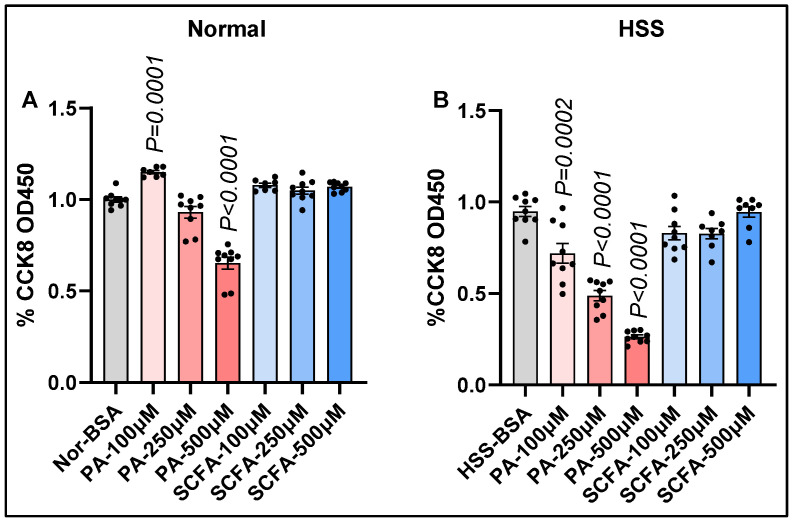
Differential effects of palmitate and short-chain fatty acids on normal vs. ischemic EC proliferation and survival. (**A**,**B**) CCK8 cell proliferation/survival assay in (**A**) normal, (**B**) hypoxia serum-starved (HSS) mSkVECs treated with BSA (vehicle control), palmitate (PA: 100 µM, 250 µM, 500 µM), or short-chain fatty acids (SCFAs: 100 µM, 250 µM, 500 µM) dose-dependently for 24 h. *n* ≥ 7 biological replicates. One-way ANOVA with Dunnett’s post-test. Mean ± SEM.

To determine the role of palmitate in vascular dysfunction, we treated normal or hypoxia serum-starved (HSS) mSkVECs [14] with palmitate dose-dependently [12] and examined the EC functions, including proliferation/survival by cell counting kit-8 (CCK8), angiogenic capacity on growth factor-reduced Matrigel (GFRM), and barrier integrity by trans-endothelial electrical resistance (TEER). ECs treated with BSA (*v*/*v*, vehicle control for palmitate) and short-chain fatty acids (SCFAs) were used as comparators for palmitate-treated ECs. While treatment of normal ECs with 100 µM palmitate significantly increased EC proliferation (*p* = 0.02), treatment with 250 µM or 500 µM had no effect or significantly decreased EC proliferation (*p* < 0.0001, Figure 1A) vs. BSA, respectively. In contrast, SCFAs did not affect normal EC proliferation at any concentration. HSS ECs treated with palmitate significantly decreased EC survival dose-dependently (100 µM: *p* = 0.03, 250 µM: *p* < 0.0001, 500 µM: *p* < 0.0001, Figure 1B). However, SCFAs did not affect HSS EC survival vs. control at any doses (Figure 1B).

### 3.2. Palmitate Inhibits, Whereas SCFAs Promote, Ischemic EC Angiogenic Capacity

**Figure 2:**
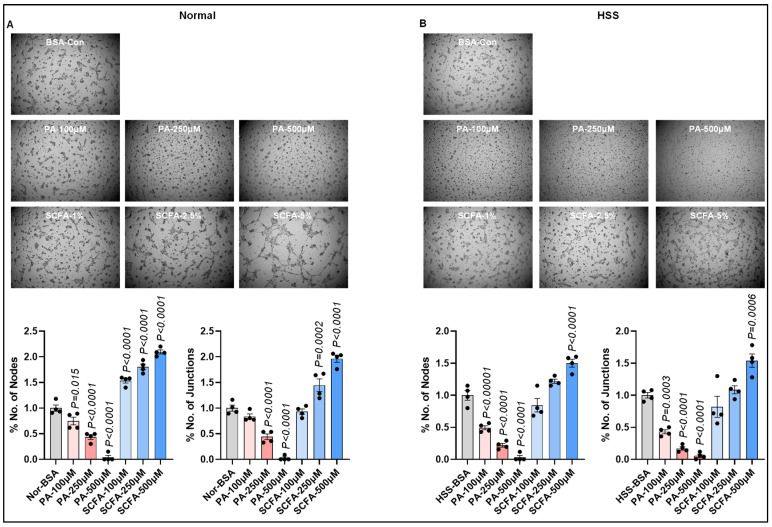
Angiogenic effects of palmitate and short-chain fatty acids on normal vs. ischemic ECs. In vitro tube formation assay of (**A**) normal, (**B**) HSS mSkVECs treated with BSA, palmitate (100 µM, 250 µM, 500 µM), or short-chain fatty acids (SCFAs: 100 µM, 250 µM, 500 µM) dose-dependently for 24 h on growth factor-reduced Matrigel. *n* = 4 biological replicates. One-way ANOVA with Dunnett’s post-test. Mean ± SEM.

In the normal ECs, while palmitate dose-dependently decreased their angiogenic capacity (%junctions: 250 µM: *p* < 0.0001, 500 µM: *p* < 0.0001; %nodes: 250 µM: *p* = 0.0002, 500 µM: *p* < 0.0001, Figure 2A), SCFAs significantly induced their angiogenic capacity dose-dependently (%junctions-2.5%SCFA: *p* = 0.0002, 5%SCFA: *p* < 0.0001; %nodes-1%SCFA: *p* = 0.004, 2.5%SCFA: *p* < 0.0001, 5%SCFA: *p* < 0.0001) vs. BSA (Figure 2A). Similarly, in HSS ECs, palmitate dose-dependently inhibited their angiogenic capacity (%junctions-100 µM: *p* < 0.0001, 250 µM: *p* < 0.0001, 500 µM: *p* < 0.0001, %nodes-100 µM: *p* = 0.0002, 250 µM: *p* < 0.0001, 500 µM: *p* < 0.0001, Figure 2B), whereas 5% SCFAs significantly increased their angiogenic capacity (%junctions: *p* = 0.0001, %nodes: *p* = 0.0002, Figure 2B). Consistent with the ability of SCFAs to induce HSS ECs’ angiogenic capacity, HSS ECs treated with SCFAs showed a significant increase in AKT activation (pAKT/AKT) vs. BSA (*p* < 0.0001, Supplementary Figure S1)

### 3.3. Palmitate Decreases, Whereas SCFAs Preserve, Ischemic EC Vascular Integrity

**Figure 3:**
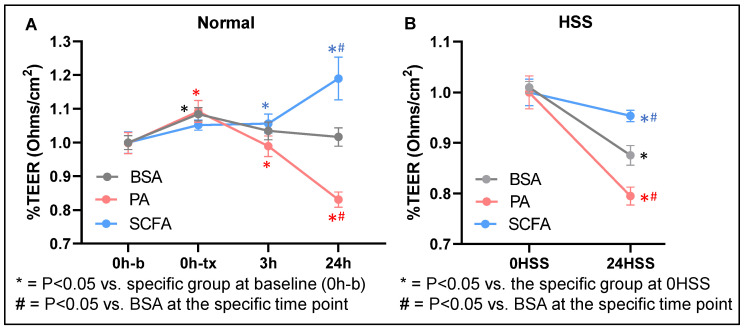
Vascular permeability effects of palmitate and short-chain fatty acids on normal vs. ischemic ECs. (**A**,**B**) Trans-endothelial electrical resistance (TEER, Ohms/cm^2^, presented as % change (denoted as 1) from 0 h timepoint of specific group. 0 h b: 0 h base line (before treatment), 0 h tx: 0 h timepoint immediately after treatment) of (**A**) normal, (**B**) HSS mSkVECs treated with BSA, 250 µM-palmitate, or SCFAs 500 µM for 24 h. *n* = 4 biological replicates. Repeated measures ANOVA with Bonferroni select pair comparison. *p* < 0.05 is considered significant. Mean ± SEM.

**Figure 4:**
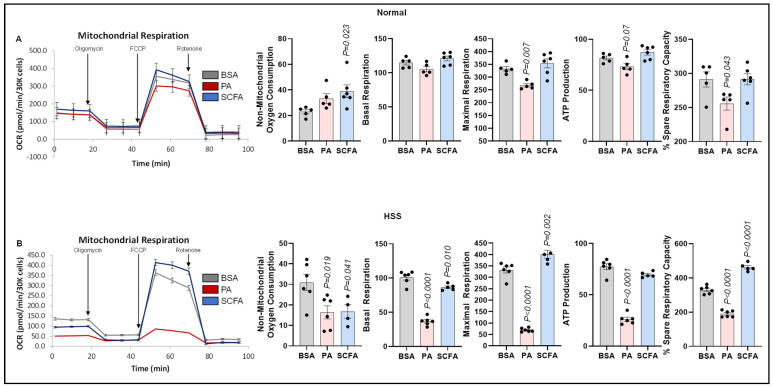
Effects of palmitate and short-chain fatty acids on mitochondrial respiration in normal vs. ischemic ECs. (**A**,**B**) Seahorse mitostress test in (**A**) normal, (**B**) HSS mSkVECs treated with BSA (*n* = 5 biological replicates), palmitate (PA-250 µM, *n* = 6 biological replicates), or short-chain fatty acids (SCFAs-500 µM, *n* = 6 biological replicates) for 24 h. One-way ANOVA with Dunnett’s post-test. Mean ± SEM.

Since palmitate showed a detrimental effect on normal and HSS EC survival and angiogenic capacity at the highest 500 µM concentration, we used a 250 µM palmitate concentration for measuring the EC barrier integrity. While ECs treated with palmitate displayed significantly decreased normal EC barrier integrity (24 h: *p* = 0.023, Figure 3A), SCFA treatment significantly increased normal EC barrier integrity (24 h: *p* = 0.032, Figure 3A). In HSS ECs, palmitate further aggravated the loss of the HSS EC barrier integrity (24HSS: *p* = 0.027), whereas SCFAs showed a modest but significant increase in ischemic EC barrier integrity compared to the control (24HSS: *p* = 0.033, Figure 4B). Consistent with the changes in the ischemic EC barrier, HSS mSkVECs treated with PA showed a significant decrease in ZO-1 (*p* = 0.048), VE-Cadherin (*p* = 0.045), and Claudin-5 (*p* < 0.001), with no significant difference in β-catenin levels compared to the control (Supplementary Figure S2). On the contrary, SCFAs significantly induced ZO-1 (*p* = 0.022), with no significant differences in VE-Cadherin or β-catenin levels compared to the controls (Supplementary Figure S2). Even though SCFAs significantly decreased the Claudin-5 levels compared to the control (*p* = 0.0016), the levels of ZO-1 were significantly higher compared to PA-treated HSS mSkVECs (*p* = 0.048, Supplementary Figure S2).

### 3.4. SCFAs Induce Distinct Metabolic Signatures from Palmitate and LCFAs in Normal and HSS ECs

#### 3.4.1. Mitochondrial Respiration

Since fatty acids undergo β-oxidation-mediated breakdown in mitochondria [10], we next examined the role of palmitate and SCFAs in regulating mitochondrial respiration/OxPhos. The Seahorse mitostress assay showed that palmitate modestly but significantly decreased maximal respiration (*p* = 0.007) and spare respiratory capacity (*p* = 0.043), and numerically decreased ATP production (*p* = 0.07), but not basal respiration in normal ECs vs. control (Figure 4A). SCFAs induced non-mitochondrial respiration but not basal respiration, maximal respiration, ATP production, or spare respiratory capacity in normal ECs. In HSS ECs, both palmitate (*p* = 0.019) and SCFAs (*p* = 0.041) significantly decreased non-mitochondrial respiration compared to the controls. However, while palmitate significantly decreased basal respiration (*p* < 0.0001), maximal respiration (*p* < 0.0001), ATP production (*p* < 0.0001) and spare respiratory capacity (*p* < 0.0001) vs. the controls, SCFA treatment only showed a modest but significant decrease in basal respiration (*p* = 0.01), whereas it significantly increased maximal respiration (*p* = 0.002) with a concurrent increase in spare respiratory capacity (*p* < 0.0001) but with no significant difference in ATP production vs. the control (Figure 4B).

Western blot analysis showed no significant difference in the SDHA levels between palmitate-treated HSS ECs vs. the controls. However, SCFAs significantly increased the levels of SDHA in HSS ECs compared to the control (Supplementary Figure S3A). Furthermore, Western blot analysis of the mitochondrial OxPhos complex showed no significant differences in complex-V (ATP synthase F1 subunit alpha (ATP5A)) or complex-IV (Mitochondrially Encoded Cytochrome C Oxidase I (MTCO1)), but a significant increase in complex-II (Succinate Dehydrogenase Complex Iron-Sulfur Subunit B (SDHB)) in HSS ECs treated with SCFAs compared to the control (Supplementary Figure S4). Representative proteins from complex-I (NADH:Ubiquinone Oxidoreductase Subunit B8 (NDUFB-8)) and complex-III (Ubiquinol-cytochrome c reductase core protein 2 (UQCRC2)) were too low to be detected within the Western blot of HSS ECs (Supplementary Figure S4).

To confirm whether mitochondrial respiration is needed for SCFA-induced angiogenic capacity in ischemic ECs, an in vitro tube formation assay on GFRM was performed on HSS ECs treated with OxPhos uncoupler carbonyl cyanide m-chlorophenylhydrazone (CCCP) (dose dependently, 1 µM or 5 µM) in the presence or absence of SCFAs. The in vitro angiogenesis assay showed that CCCP at a 5 µM concentration significantly decreased the angiogenic capacity of HSS ECs (% nodes: Con vs. CCCP (5 µM)-*p* < 0.0001, % junctions: Con vs. CCCP (5 µM)-*p* < 0.0001). SCFA treatment was not able to restore the angiogenic capacity of HSS ECs treated with CCCP (Supplementary Figure S5).

#### 3.4.2. Fatty Acid Oxidation

**Figure 5:**
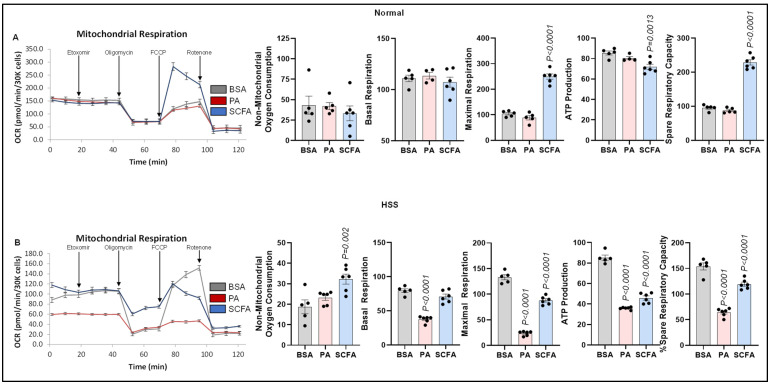
Effects of palmitate and SCFAs on normal vs. ischemic EC β-oxidation. (**A**,**B**) Seahorse basal fatty acid oxidation test (that uses CPT1 inhibitor without exogenous/additional palmitate in the Seahorse assay medium) in (**A**) normal, (**B**) HSS mSkVECs treated with BSA (*n* = 5 biological replicates), palmitate (PA-250 µM, *n* = 6 biological replicates), or short-chain fatty acids (SCFAs 500 µM, *n* = 6 biological replicates) for 24 h. One-way ANOVA with Dunnett’s post-test. *p* < 0.05 is considered significant. Mean ± SEM.

While palmitate relies on the carnitine shuttle system [10], SCFAs can directly diffuse into mitochondria for β-oxidation [11]. Thus, we next determined whether the changes in mitochondrial respiration induced by palmitate vs. SCFA treatment are due to differential substrate oxidation in normal and HSS ECs. Since ECs are already treated with palmitate and SCFAs, a Seahorse basal-FAO assay was performed. The FAO assays showed no significant effect of palmitate on non-mitochondrial oxygen consumption, basal respiration, maximal respiration, ATP production, or spare respiratory capacity compared to the controls in normal ECs (Figure 5A). Similarly, SCFAs also did not show any significant effect on non-mitochondrial oxygen consumption, basal respiration, or maximal respiration compared to the controls in normal ECs. However, a significant decrease in ATP production (*p* = 0.0013) with a concomitant increase in spare respiratory capacity (*p* < 0.0001) was observed in normal ECs treated with SCFAs compared to controls (Figure 5A).

In HSS ECs, the FAO assays showed that palmitate did not significantly affect non-mitochondrial oxygen consumption but significantly decreased basal respiration (*p* < 0.0001), maximal respiration (*p* < 0.0001), ATP production (*p* < 0.0001), and spare respiratory capacity (*p* < 0.0001) compared to the controls (Figure 5B). On the other hand, while SCFAs significantly induced non-mitochondrial oxygen consumption (*p* = 0.002), no significant changes in basal respiration was observed (Figure 5B). Interestingly, consistent with the changes in FAO induced by palmitate, SCFAs also significantly decreased maximal respiration (*p* < 0.0001), ATP production (*p* < 0.0001), and spare respiratory capacity (*p* < 0.0001) compared to the controls (Figure 5B). Inhibiting CPTs not only aggravated the loss of mitochondrial respiration in palmitate-treated HSS ECs but also inhibited SCFA-induced mitochondrial respiration, suggesting that the ability of SCFAs to induce mitochondrial respiration in HSS ECs is also dependent on CPTs. Intriguingly, while Western blot analysis showed no significant difference in CPT2 levels between palmitate-treated HSS-ECs vs. controls, SCFA-treated HSS ECs displayed a significant increase in CPT2 levels compared to the controls (Supplementary Figure S3B)

#### 3.4.3. Glycolysis

**Figure 6:**
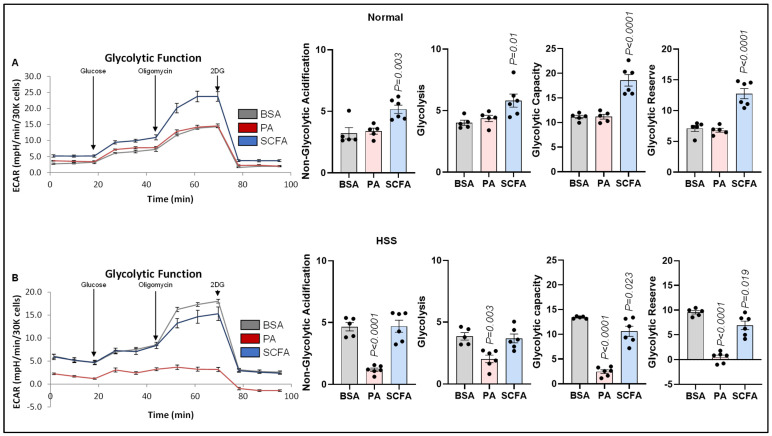
Effect of palmitate and short-chain fatty acids on normal and ischemic EC glycolysis. (**A**,**B**) Seahorse glycolysis stress test in (**A**) normal, (**B**) HSS mSkVECs treated with BSA (*n* = 5 biological replicates), palmitate (PA-250 µM, *n* = 5 biological replicates), or short-chain fatty acids (SCFAs 500 µM, *n* = 6 biological replicates) for 24 h. One-way ANOVA with Dunnett’s post-test. Mean ± SEM.

Since ECs primarily rely on glycolysis to meet their metabolic demand [18], we examined the compensatory effect of palmitate and SCFA β-oxidation on normal and HSS EC glycolysis. The Seahorse glycolysis stress assay showed no significant effect of palmitate on normal EC non-glycolytic acidification, glycolysis, glycolytic capacity, or glycolytic reserve (Figure 6A). However, SCFAs significantly increased non-glycolytic acidification (*p* = 0.003), glycolysis (*p* = 0.01), glycolytic capacity (*p* < 0.0001), and glycolytic reserve (*p* < 0.0001) vs. the control (Figure 6A). In HSS ECs, palmitate significantly decreased non-glycolytic acidification (*p* < 0.0001), glycolysis (*p* = 0.003), glycolytic capacity (*p* < 0.0001), and glycolytic reserve (*p* < 0.0001) vs. the control (Figure 6B). SCFAs did not affect non-glycolytic acidification or glycolysis but significantly decreased glycolytic capacity (*p* = 0.023) and glycolytic reserve (*p* = 0.019) vs. the controls (Figure 6B).

Consistent with our recent data indicating that increased PFKFB3 expression inhibits the angiogenic capacity of ischemic ECs [15], Western blot analysis showed a significant increase in PFKFB3 levels in PA-treated HSS ECs (*p* = 0.026, Supplementary Figure S3C) without any significant differences in hexokinase-2 (HK2) levels (Supplementary Figure S3D) compared to the controls. On the contrary, SCFAs significantly induced PFKFB3 (*p* < 0.0001, Supplementary Figure S3C) and HK2 (*p* = 0.025, Supplementary Figure S3D) levels vs. the controls.

## 4. Discussion

Palmitate (C16:0), one of the most abundant circulating saturated fatty acids, is physiologically important for lipid metabolism [19]. However, excessive palmitate levels are associated with endothelial dysfunction, a key early event in cardiovascular pathologies [20]. While the specific levels of palmitic acid in diabetic PAD patients were not available, in a study by Trombetta et al. [21], the serum levels of non-esterified fatty acids in diabetic patients were observed to be ~0.45 g/L. The authors indicate that the palmitic acid constituted ~30% of the total NEFAs, thereby presenting the circulating palmitic acid levels to be ~0.54 mM. Furthermore, a study by Wang et al. [22] showed that doses higher than 0.4 mM palmitate cause extensive macrophage cell death, which is consistent with our observations. Taken together, the 250 μM concentration of palmitate used in our in vitro studies falls within the range observed in human cardiovascular diseases and in our previous in vitro experiments characterizing the deleterious effects of palmitate. Consistent with our earlier study, palmitate dose-dependently decreased ischemic EC survival and angiogenic capacity [12], recapitulating diabetic PAD conditions in vitro. The contribution of palmitate to EC functional impairment as well as the pathogenesis of multiple cardiovascular diseases has been demonstrated previously [5,23,24]. In vitro, studies have shown that palmitate induces inflammatory cytokine production, including TNF-α, IL-6, MCP1, and LDL1, in macrophages while decreasing NO production in ECs [25,26]. Importantly, elevated palmitate levels have been positively associated with atherosclerotic plaque vulnerability and major cardiovascular adverse events in T2D [22]. Furthermore, previous studies have indicated that palmitate, but not palmitoleic acid, induces insulin resistance [27]; palmitate, but not stearic acid, inhibits NO production [28]; and finally, palmitate inhibits, whereas oleate promotes, EC proliferation [29]. These studies implicate palmitate-mediated endothelial injury as a central link between dietary saturated fat, metabolic disorders, and cardiovascular disease.

Metabolic disturbances, particularly those associated with elevated free fatty acids, compromise endothelial homeostasis [30]. Palmitate increases the accumulation of ceramides and diacylglycerols, which disrupt cell signaling and trigger apoptosis [31]. This, coupled with endoplasmic reticulum (ER) stress and activation of the unfolded protein response (UPR), leads to the expression of pro-apoptotic mediators, such as CHOP, and culminates in cell death [32]. A major contributor to palmitate-induced vascular dysfunction is oxidative stress. Palmitate increases mitochondrial and NADPH oxidase-derived reactive oxygen species (ROS), decreasing endothelial nitric oxide synthase (eNOS) activity and nitric oxide (NO) bioavailability. The resulting NO deficit impairs vasodilation and promotes inflammation. Furthermore, palmitate activates NF-κB and JNK signaling, enhancing the expression of adhesion molecules (VCAM-1, ICAM-1) and inflammatory cytokines (IL-6, TNF-α), thereby facilitating leukocyte recruitment and atherogenesis [26]. Prolonged exposure to palmitate increases endothelial permeability and disrupts tight junction proteins such as VE-Cadherin and Claudin-5 [33]. This barrier dysfunction contributes to vascular leakage and early atherosclerotic lesion formation. Together, palmitate induces oxidative, inflammatory, and metabolic stress pathways that converge on impaired vascular reactivity. Therefore, therapeutic interventions to minimize palmitate levels or block palmitate-induced reactive oxygen species generation, ceramide synthesis, and/or inflammatory signaling in ischemic ECs hold great potential to rescue vascular dysfunction and preserve endothelial integrity in ischemic cardiovascular diseases.

While data on the circulating SCFA levels in PAD or diabetic PAD patients is very limited, a recent study by Muradi et al. [9] showed that the median fecal SCFA content in diabetic PAD patients was ~100 mM (acetate 59 mM, propionate 20.38 mM, 20.66 mM, and Valerate 2.93 mM). Even though the fecal SCFA content may not be the best parameter for comparing/titrating in vitro doses, the 500 µM concentration used in our study is lower than the observed SCFA content in diabetic PAD patients. In contrast with palmitate, SCFAs did not show any significant effect on normal EC proliferation or ischemic EC survival. However, SCFA treatment significantly increased the angiogenic capacity of both normal and ischemic ECs vs. the controls. Consistently, SCFAs significantly induced AKT activation in ischemic ECs. Additionally, SCFAs improved and preserved normal and ischemic EC barrier integrity, respectively, despite enhancing ischemic EC angiogenic capacity, indicating the ability of SCFAs to drive functional angiogenesis (enhanced angiogenic capacity with preserved barrier integrity).

As ECs primarily depend on glycolysis [18] for their energy requirements, the metabolic processes regulated by palmitate and SCFAs that might mediate ischemic EC function are not fully characterized. Importantly, long-chain fatty acid ingress into mitochondria is gatekept by the carnitine shuttle system [10], whereas the smaller size of SCFAs allows them to freely diffuse into mitochondria for β-oxidation independent of the CPT transport system [11]. Consistently, in normal ECs, SCFAs induced mitochondrial respiration independent of CPT activity. However, under HSS conditions, inhibiting CPTs blocked SCFA-driven induction of mitochondrial respiration, indicating that the ability of SCFAs to induce ischemic EC mitochondrial respiration is partly dependent on the CPT system. Accordingly, SCFAs induced CPT2 levels in HSS ECs. Consistent with our data, a recent report by Hao et al. [34] showed that butyrate increased the CPT1A levels/activity in T-cells to facilitate FAO. Taken together, these data present a novel mechanism of CPT-mediated β-oxidation of SCFAs in HSS ECs, which is essential to induce ischemic angiogenesis.

Furthermore, our recent studies have shown that ischemia increases PFKFB3 levels, leading to maladaptive glycolysis that inhibits ischemic angiogenesis [14,17]. While both palmitate and SCFAs induced PFKFB3/glycolysis, palmitate-mediated glycolysis did not accompany elevated mitochondrial respiration, indicating defective glycolysis–OxPhos coupling. On the contrary, SCFAs induced (1) PFKFB3 levels without a net increase in glycolysis; and (2) a significant increase in mitochondrial respiration/SDH in ischemic ECs. These data indicate that SCFA treatment increases glycolysis–OxPhos coupling in ischemic ECs, resulting in no net change in glycolysis (or normalizing glycolysis), with a concomitant increase in mitochondrial respiration. Taken together, these results suggest that SCFAs increase the coupling of glycolysis and OxPhos in HSS ECs, reflecting increased PFKFB3 and SDHA/B levels necessary to induce functional ischemic angiogenesis, whereas palmitate increases PFKFB3 expression alone in HSS ECs, thereby driving maladaptive glycolysis and inhibiting ischemic angiogenesis. While previous reports have partially characterized SCFA regulation of mitochondrial respiration, a direct role of SCFAs in driving EC glycolysis has not been elucidated. Hence, it is unclear whether increased glycolysis in SCFA-treated ischemic ECs is due to an improvement in overall ischemic EC metabolic health or a direct effect on ischemic EC glycolysis, necessitating further investigation. Nevertheless, our data suggest a novel role of SCFAs in controlling metabolic checkpoints in glycolysis–OxPhos coupling in ischemic ECs.

While much of the research has been focused on epithelial and immune cells in the gut, growing evidence indicates that SCFAs influence the vascular endothelium through direct and indirect mechanisms that act to modulate EC function, permeability, oxidative stress, and inflammation. In in vitro EC models, SCFAs have been shown to increase eNOS activation and NO production, elevate NADPH oxidase and mitochondria-derived ROS, attenuate expression of adhesion molecules and inflammasome activation, and support mitochondrial and endothelial barrier integrity [35,36]. In rat aortic ECs and intact aortic rings, butyrate and acetate (but not propionate) reversed angiotensin II (Ang II)-induced EC dysfunction [35]. Specifically, SCFAs restored NO production, enhanced vasodilator-stimulated phosphoprotein phosphorylation at Ser239 [35], reduced intracellular ROS, and improved acetylcholine-induced relaxation. Furthermore, butyrate, but not acetate or propionate, reduced NLRP3 inflammasome formation in ECs exposed to pro-atherogenic stimuli and reduced carotid neointima formation in mouse models, suggesting differential SCFA effects on vascular inflammation [37]. More importantly, in human brain microvascular EC models exposed to Ang II, SCFA treatment rescued heme oxygenase-2 expression, normalized mitochondrial membrane potential/respiration, reduced mitochondrial ROS (specifically H_2_O_2_), and restored eNOS phosphorylation [38], linking SCFA effects to mitochondrial/endothelial crosstalk. These actions position SCFAs as potential key mediators of both gut–vascular communication and cardiovascular health. Further preclinical and clinical research is required to determine whether modulation of SCFA levels and/or signaling can be leveraged to ameliorate endothelial dysfunction and the cardiovascular disease risk.

## 5. Future Directions

Despite promising in vitro findings, translation of the identified SCFA roles in EC function to human pathologies remains limited. The differential effects of individual SCFAs also warrant deeper investigation. Future studies should employ preclinical PAD models to address microvascular vs. macrovascular specificity and the therapeutic feasibility of targeting SCFAs for PAD treatment. Furthermore, whether SCFA treatment can overcome the detrimental effects of palmitate in ischemic vasculature needs further investigation.

## Data Availability

All data generated or analyzed during this study are included in this published article and its Supplementary Information. Any additional data can be made available by the corresponding author upon reasonable request.

## Supplementary Materials

The following supporting information can be downloaded at https://www.mdpi.com/article/10.3390/cells15060493/s1, Supplementary methods, Supplementary Figure S1: Effect of palmitate and short chain fatty acids on AKT activation in ischemic-ECs. Western blot analysis of pAKT/AKT in ischemic-ECs treated with BSA, palmitate (250 µM), or SCFAs (500 µM). *n* = 4. One-way ANOVA with Dunnett’s post-test. *p* < 0.05 considered significant. Mean ± SEM, Supplementary Figure S2: Effect of palmitate and short chain fatty acids on junctional molecule levels in ischemic-EC. Western blot analysis of ZO-1, VE-Cadherin, β-catenin and Claudin-5 in ischemic-ECs treated with BSA, palmitate (250 µM), or SCFAs (500 µM). *n* = 4. One-way ANOVA with Dunnett’s post-test. *p* < 0.05 considered significant. Mean ± SEM, Supplementary Figure S3: Differential effects of palmitate, and short-chain fatty acids on metabolic regulators. Western blot analysis of (Hexokinase-2), 6-phosphofructo-2-kinase/fructose-2,6-biphosphatase 3 (PFKFB3), Succinate dehydrogenase (SDH), and Carnitine palmitoyl transferase-1 (CPT1) in ischemic ECs treated with BSA, palmitate (250 µM), or SCFAs (500 µM). *n* = 4. One-way ANOVA with Dunnett’s post-test. *p* < 0.05 considered significant. Mean ± SEM, Supplementary Figure S4: Effect of short chain fatty acids on mitochondrial complex protein levels in ischemic-ECs. Western blot analysis of mitochondrial complex proteins in ischemic-ECs treated with BSA, or SCFAs (500 µM). *n* = 4. Unpaired T-Test. *p* < 0.05 considered significant. Mean ± SEM, Supplementary Figure S5: Role of mitochondrial respiration on angiogenic capacity induced by short chain fatty acids in ischemic-ECs. In vitro tube formation assay of ischemic-ECs treated with CCCP dose dependently (1 μM or 5 μM) in the presence or absence of SCFAs (500 μM) on growth factor reduced Matrigel. *n* = 6. One Way ANOVA with Dunnett’s post-test. *p* < 0.05 considered significant. Mean ± SEM.

## Abbreviations

mSkVECs	Mouse skeletal muscle microvascular endothelial cells
ECs	Endothelial cells
HSS	Hypoxia serum starvation
TEER	Trans-endothelial electrical resistance
GFRM	Growth factor-reduced Matrigel
SCFAs	Short-chain fatty acids
PA	Palmitic acid
BSA	Bovine serum albumin
