# Supplementary material for: Short-Chain Fatty Acids and Palmitate Induce Distinct Metabolic and Phenotypic Signatures in Normal and Ischemic Skeletal Muscle Microvascular Endothelial Cells
Source: Cells. 2026 Mar 10;15(6):493. doi: 10.3390/cells15060493 (PMC13025840; doi:10.3390/cells15060493)
Supplement: Supplementary file 1 [file cells-15-00493-s001.zip › SCFAs-Palmitate-mSkVECs-Cells-Supplemental Figure legend.pdf]

Supplementary Methods

Western blot analysis

Cells were lysed in RIPA lysis buffer (Cat No: sc-24948A, Santa Cruz Biotechnology). Total protein was estimated using a BCA kit. At least 10-15µg of cell lysate, was resolved on SDS-PAGE, transferred onto nitrocellulose membrane, and western blotted for Hexokinase-2 (HK2), Cat No: 2867, Cell signaling and Technology), 6-phosphofructo-2-kinase/fructose-2,6-bisphosphatase 3 (PFKFB3, Cat No: EPR12594, Abcam), Carnitine palmitoyltransferase II (CPT2, Cat NO: A0567, AbClonal), Succinate Dehydrogenase Complex Flavoprotein Subunit A (SDHA, Cat No: 5839, Cell signaling and Technology), and Actin (Cat No: AC004, AbClonal). Western blot images were captured using the iBright Imaging System. Each sample was considered as a biological replicate, and an n of 4 biological replicate samples were used for western blot analysis. Each Western blot was repeated twice, and the best representative images were presented.

Supplementary Figure Legend

Supplementary Figure-S1: Effect of palmitate and short chain fatty acids on AKT activation in ischemic-ECs. Western blot analysis of pAKT/AKT in ischemic-ECs treated with BSA, palmitate (250μM), or SCFAs (500μM). n=4. One-way ANOVA with Dunnett's post-test. P<0.05 considered significant. Mean±SEM

Supplementary Figure-S2: Effect of palmitate and short chain fatty acids on junctional molecule levels in ischemic-EC. Western blot analysis of ZO-1, VE-Cadherin, β-catenin and Claudin-5 in ischemic-ECs treated with BSA, palmitate (250μM), or SCFAs (500μM). n=4. One-way ANOVA with Dunnett's post-test. P<0.05 considered significant. Mean±SEM

Supplementary Figure-S3: Differential effects of palmitate, and short-chain fatty acids on metabolic regulators. Western blot analysis of (Hexokinase-2), 6-phosphofructo-2-kinase/fructose-2,6-biphosphatase 3 (PFKFB3), Succinate dehydrogenase (SDH), and Carnitine palmitoyl transferase-1 (CPT1) in ischemic ECs treated with BSA, palmitate (250μM), or SCFAs (500μM). n=4. One-way ANOVA with Dunnett's post-test. P<0.05 considered significant. Mean±SEM

Supplementary Figure-S4: Effect of short chain fatty acids on mitochondrial complex protein levels in ischemic-ECs. Western blot analysis of mitochondrial complex proteins in ischemic-ECs treated with BSA, or SCFAs (500μM). n=4. Unpaired T-Test. P<0.05 considered significant. Mean±SEM

Supplementary Figure-S5: Role of mitochondrial respiration on angiogenic capacity induced by short chain fatty acids in ischemic-ECs. *In vitro* tube formation assay of ischemic-ECs treated with CCCP dose dependently (1μM or 5 μM) in the presence or absence of SCFAs (500μM) on growth factor reduced Matrigel. n=6. One Way ANOVA with Dunnett's post-test. P<0.05 considered significant. Mean±SEM
