# Supplementary figures and images for: Short-Chain Fatty Acids and Palmitate Induce Distinct Metabolic and Phenotypic Signatures in Normal and Ischemic Skeletal Muscle Microvascular Endothelial Cells
Source: Cells. 2026 Mar 10;15(6):493. doi: 10.3390/cells15060493 (PMC13025840; doi:10.3390/cells15060493)

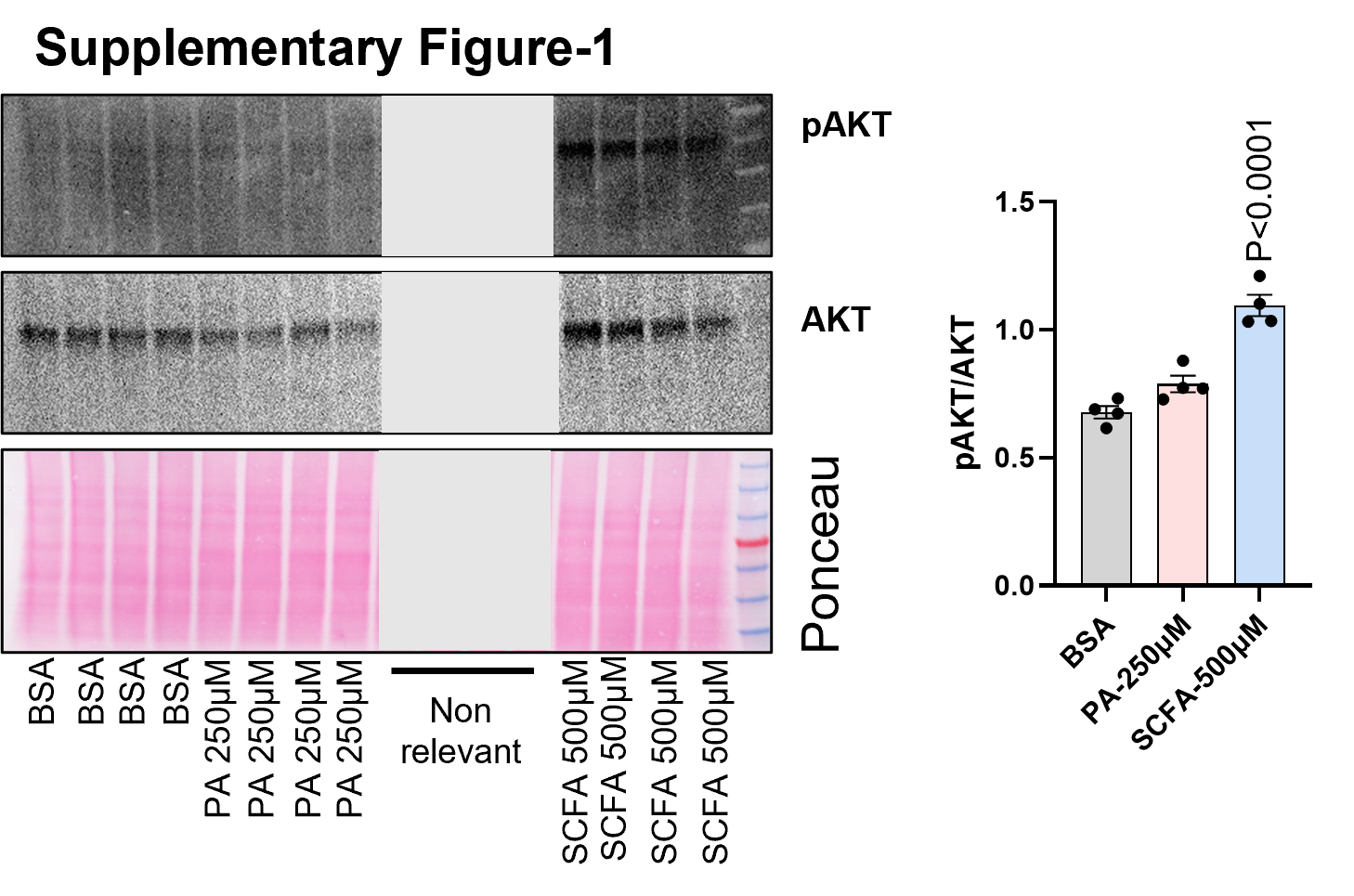

Supplement: Supplementary file 1 [file cells-15-00493-s001.zip › Figure S1.tif]

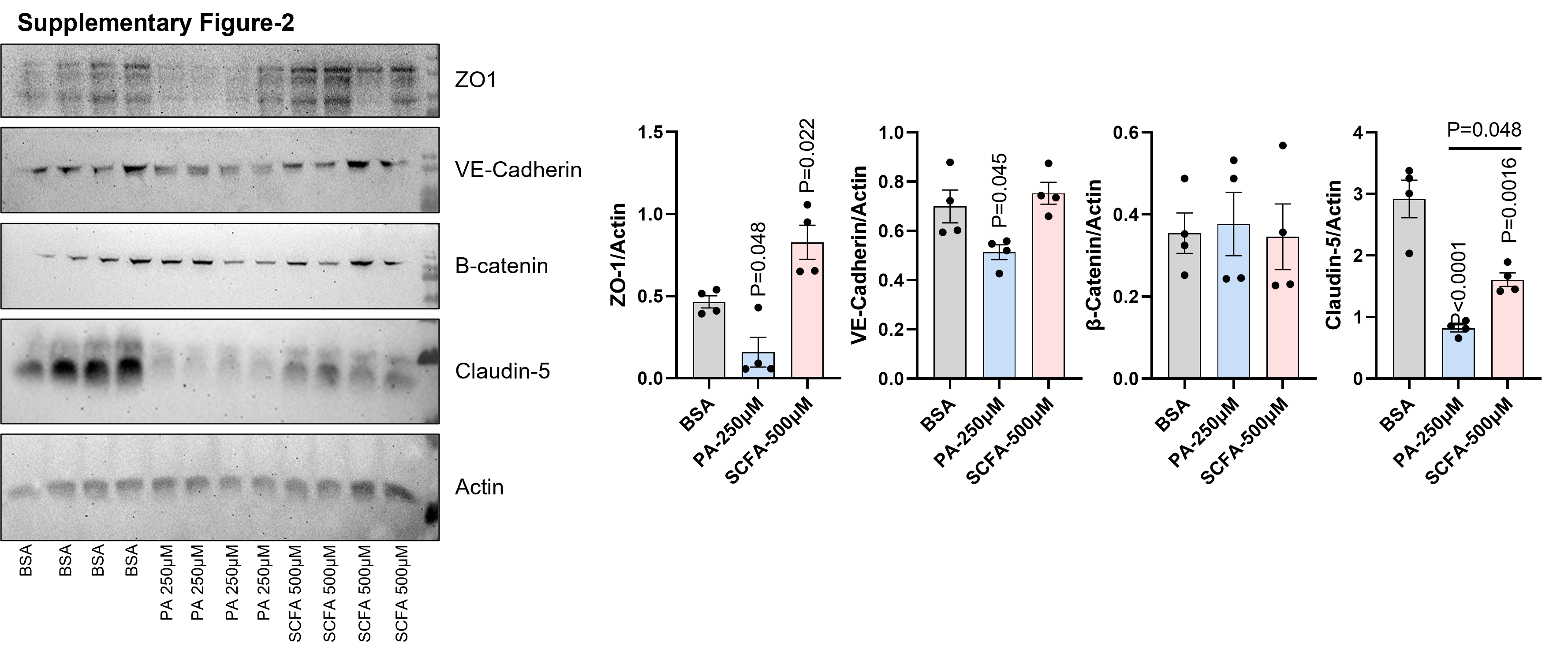

Supplement: Supplementary file 1 [file cells-15-00493-s001.zip › Figure S2.tif]

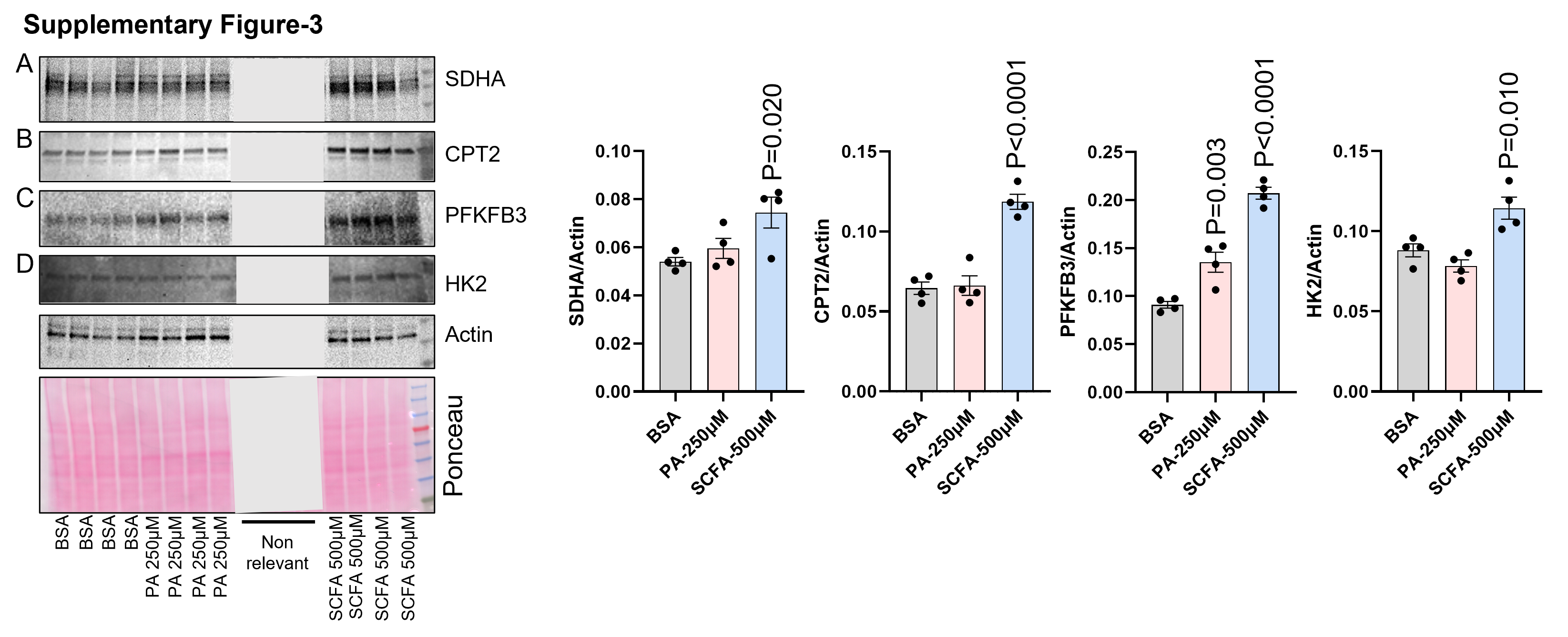

Supplement: Supplementary file 1 [file cells-15-00493-s001.zip › Figure S3.tif]

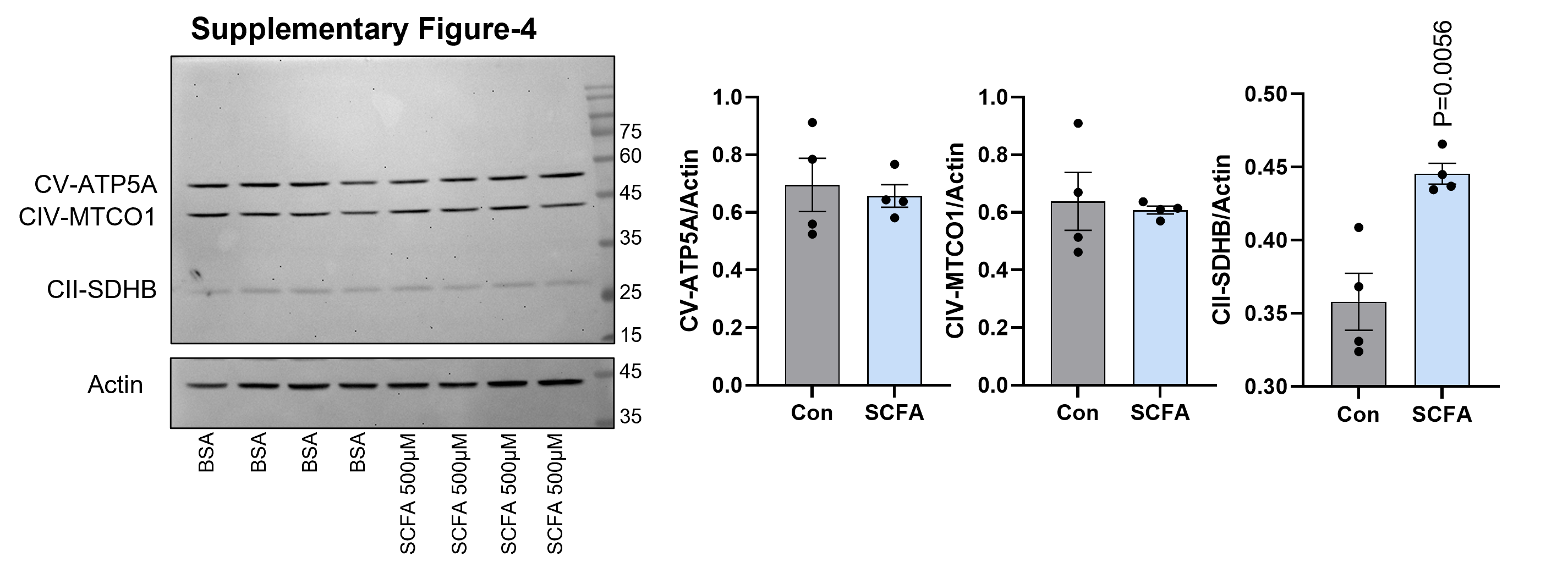

Supplement: Supplementary file 1 [file cells-15-00493-s001.zip › Figure S4.tif]

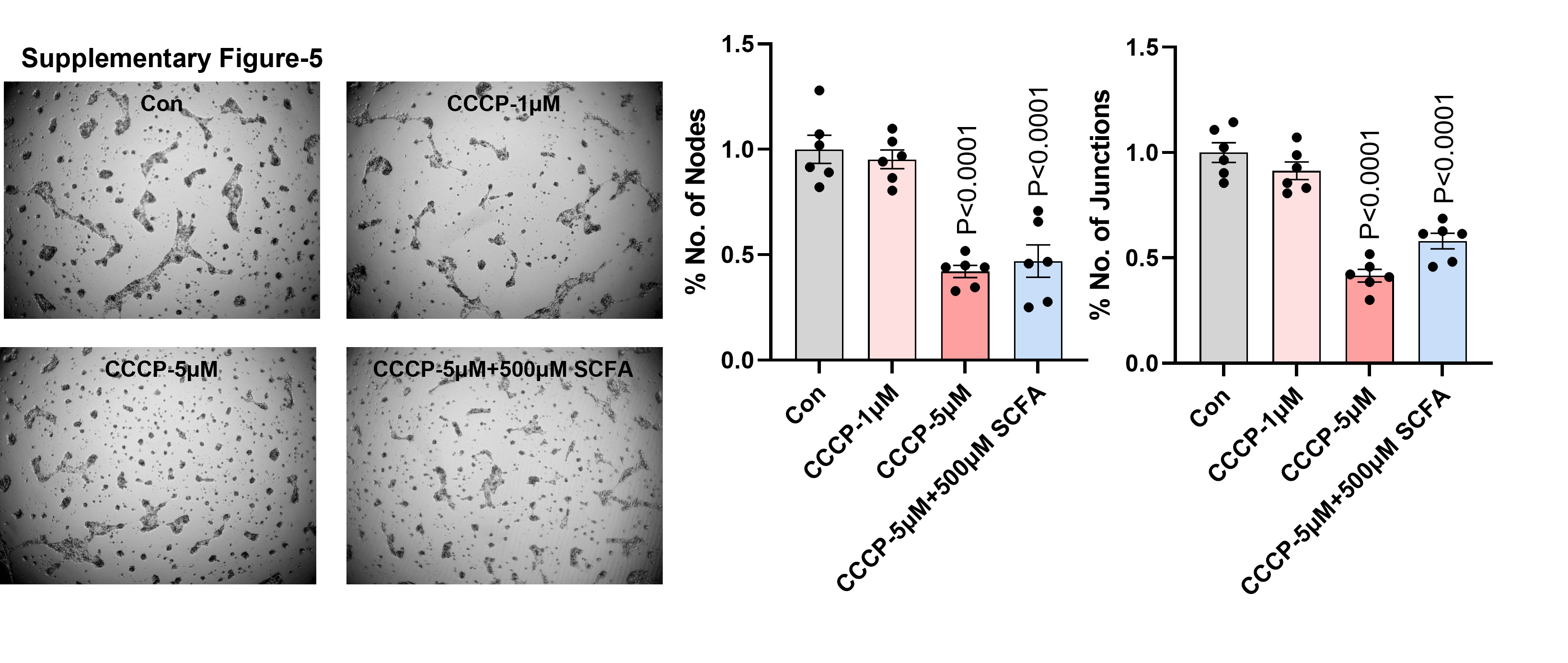

Supplement: Supplementary file 1 [file cells-15-00493-s001.zip › Figure S5.tif]
